# Posttranslational isoprenylation of tryptophan in bacteria

**DOI:** 10.3762/bjoc.13.37

**Published:** 2017-02-22

**Authors:** Masahiro Okada, Tomotoshi Sugita, Ikuro Abe

**Affiliations:** 1 Graduate School of Pharmaceutical Sciences, The University of Tokyo, Bunkyo-ku, Tokyo 113-0033, Japan

**Keywords:** *Bacillus subtilis*, isoprenylation, post-translational modification, quorum sensing, tryptophan

## Abstract

Posttranslational isoprenylation is generally recognized as a universal modification of the cysteine residues in peptides and the thiol groups of proteins in eukaryotes. In contrast, the *Bacillus* quorum sensing peptide pheromone, the ComX pheromone, possesses a posttranslationally modified tryptophan residue, and the tryptophan residue is isoprenylated with either a geranyl or farnesyl group at the gamma position to form a tricyclic skeleton that bears a newly formed pyrrolidine, similar to proline. The post-translational dimethylallylation of two tryptophan residues of a cyclic peptide, kawaguchipeptin A, from cyanobacteria has also been reported. Interestingly, the modified tryptophan residues of kawaguchipeptin A have the same scaffold as that of the ComX pheromones, but with the opposite stereochemistry. This review highlights the biosynthetic pathways and posttranslational isoprenylation of tryptophan. In particular, recent studies on peptide modifying enzymes are discussed.

## Introduction

Posttranslational modification is the chemical modification of proteins after their translation from mRNAs to the corresponding polypeptide chains synthesized by ribosomes. Since a posttranslational modification generates a novel amino acid residue in ribosomally synthesized proteins consisting of the twenty normal amino acid residues, it increases the structural diversity of ordinary proteins. In addition, newly synthesized proteins lacking the modification often cannot perform the functions of the mature proteins. Therefore, posttranslational modifications dynamically regulate the biological activities of proteins. Novel modifications have been discovered over the last several decades, revealing numerous post-translational modification patterns, including isoprenylation [1–2]. This review will discuss the posttranslational isoprenylation of tryptophan in bacteria. In particular, this review will focus on current findings which have not been available at the time we published a review on this topic previously [3].

## Review

### Posttranslational isoprenylation of cysteine

**Figure 1:**
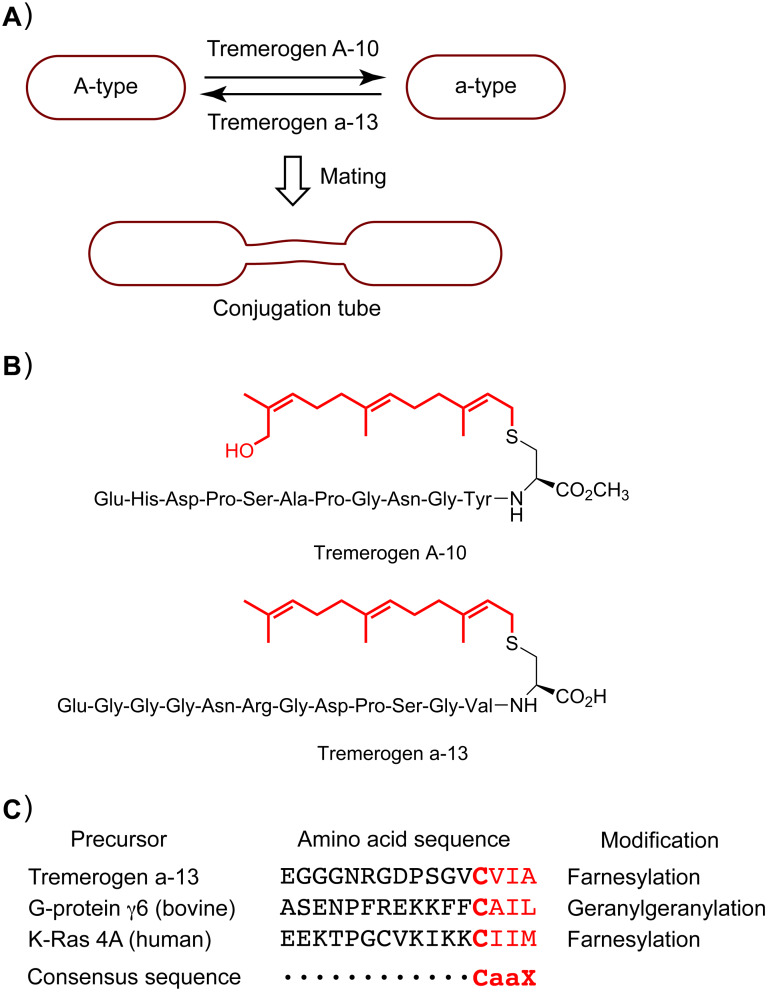
(A) Schematic representation of pheromone-induced conjugation tube formation for mating in *Tremella mesenterica*. (B) Chemical structures of tremerogens A-10 and a-13. The isoprenyl side chains are shown in red. (C) C-terminal amino acid sequences of the precursors of isoprenylated peptides and proteins. The CaaX motifs are shown in red.

Posttranslational isoprenylation is generally referred to as the farnesylation or geranylgeranylation of the thiol group of the C-terminal cysteine residue in peptides and proteins [4–7]. The isoprenylation of cysteine was first found in the peptide pheromones of basidiomycetous yeast [8–10]. Two peptide pheromones, tremerogen A-10 and tremerogen a-13, are secreted by the yeast-form haploid A-type and a-type cells of *Tremella mesenterica*, respectively (Figure 1A). Tremerogen A-10 is a decapeptide containing a hydroxyfarnesylated C-terminal cysteine methyl ester, whereas tremerogen a-13 is a tridecapeptide containing a farnesylated C-terminal cysteine (Figure 1B) [9–10]. Each pheromone causes the opposite type of cell to induce the reciprocal conjugation of the heterothallic cells, through the formation of a conjugation tube for mating. A structure–activity relationship study on tremerogen A-10 demonstrated that both the amino acid sequence and the hydrophobic side chain were essential for the initiation of the conjugation tube formation [11]. Soon thereafter, the consensus sequence for the isoprenylation of the cysteine in the precursor peptide was identified as the CaaX motif, in which "a" refers to an aliphatic amino acid and "X" refers to an appropriate amino acid, depending on the types of modifying enzymes (Figure 1C) [4–7]. Therefore, in the process of isoprenylated peptide and protein biosynthesis, the cysteine residue of the CaaX motif is isoprenylated by isoprenyltransferase, and then the last three amino acids are processed, often with methyl esterification of the resulting C-terminal isoprenylcysteine. Considering the consensus sequence, a variety of organisms may produce isoprenylated peptides and proteins. Subsequently, numerous isoprenylated peptides and proteins, such as G-proteins including the human oncogene product K-Ras, were identified from various organisms based on the consensus sequence (Figure 1C). Since the tumor growth induced by K-Ras is highly dependent on the farnesylation, the K-Ras farnesyltransferase has attracted keen attention as a target protein for anti-cancer therapy [12]. Posttranslational isoprenylation is now recognized as being universal in eukaryotes, and playing an essential role in protein functions.

### ComX pheromone

**Figure 2:**
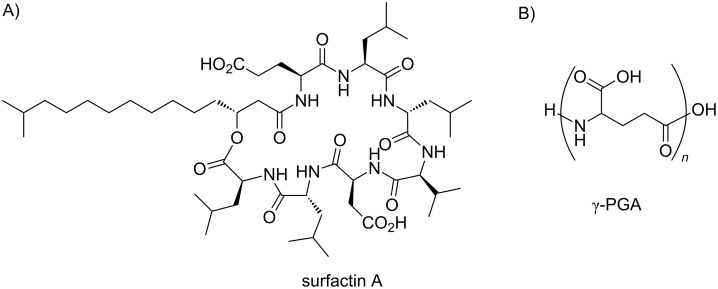
Chemical structures of (A) surfactin A and (B) poly-γ-glutamic acid.

In contrast to eukaryotes, cysteine isoprenylation has not been detected in prokaryotes. Posttranslational isoprenylation in prokaryotes was first found in a tryptophan residue of the quorum sensing pheromone from *Bacillus subtilis*, the ComX pheromone [13]. Quorum sensing is a specific gene expression system dependent on the cell density [14]. In terms of a competition for survival, the cell population density is one of the largest factors for microorganisms because of a high proliferation rate. In the quorum sensing process, bacteria constitutively secrete specific extracellular signaling molecules, called quorum sensing pheromones, to gather information about their cell population density [15–18]. Various phenomena are stimulated by an increase in the bacterial population density, or in other words, the concentration of the specific secreted pheromone. The ComX pheromone induces natural genetic competence under the control of quorum sensing in *B. subtilis*. Specifically, the ComX pheromone induces competent cell formation for DNA transformation at a high population cell density in *B. subtilis* [19–20]. In addition, the ComX pheromone promotes the production of surfactin A, a cyclic lipopeptide with antibiotic and biological surfactant activities (Figure 2A) [21–22]. Furthermore, the ComX_natto_ pheromone from *B. subtilis* subsp. *natto* contributes to the phenotypic characteristics involved in biofilm formation by *B. subtilis* subsp. *natto*, which is closely related to the *Bacillus* laboratory strains and renowned as the producer strain for the quite sticky, traditional Japanese food natto, made from fermented soybeans [23]. *B. subtilis* subsp. *natto* is obviously distinct from the other laboratory strains with respect to the biofilm formation. The biofilm mainly consists of the highly sticky poly-γ-glutamic acid (γ-PGA) polymer (Figure 2B), and the ComX_natto_ pheromone activates γ-PGA biosynthesis in *B. subtilis* subsp. *natto* at nanomolar levels [24].

**Figure 3:**
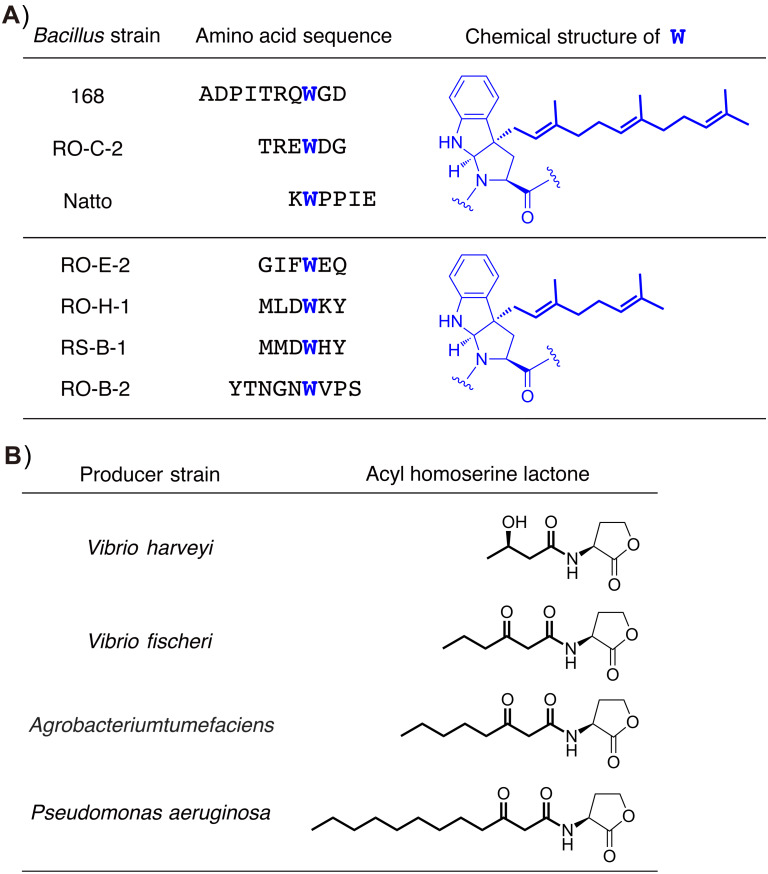
(A) Two types of posttranslational isoprenylations of ComX variants. The modified tryptophan residues are colored blue. The isoprenyl side chains are shown in boldface and colored blue. (B) Chemical structures of acyl homoserine lactones. The acyl side chains are shown in boldface.

The ComX pheromones are oligopeptides, and their amino acid sequences and lengths vary widely among *Bacillus* strains (Figure 3A) [13,21,25]. However, each ComX pheromone possesses an invariant tryptophan residue as a single common denominator, and the tryptophan residue is isoprenylated with either a geranyl or farnesyl group at the gamma position to form tricyclic skeleton that bears a newly formed pyrrolidine, which is similar to proline (Figure 3A) [26–28]. The posttranslational modification of ComX pheromones with an isoprenoid plays an essential role for specific quorum sensing responses in *B. subtilis* and related bacilli [3]. Structure–activity relationship studies on the ComX_RO-E-2_ pheromone derived from *Bacillus* strain RO-E-2, which is a hexapeptide with a geranyl-modified tryptophan residue, revealed that the exact chemical structure of the geranyl group and the absolute configurations of the tricyclic core scaffold were essential and more critical for its pheromonal activity than the amino acid sequence of the ComX_RO-E-2_ pheromone [29–32]. In addition, a previous study using a conditioned medium with *Bacillus* strains suggested that the chemical structure of the isoprenyl side chain is an influential factor of the group- (or species-) specific pheromonal activity [25]. Intriguingly, the same applies for group- (or species-) specificity in Gram negative bacteria because the chemical structure and length of the acyl side chain in acylhomoserine lactones, which are quorum sensing pheromones secreted by Gram negative bacteria, have a great effect on the group specificity (Figure 3B) [33–34]. Modifications of the lipophilic side chain in quorum sensing pheromones are probably a common strategy to acquire group specificity in bacteria.

### ComQ

**Figure 4:**
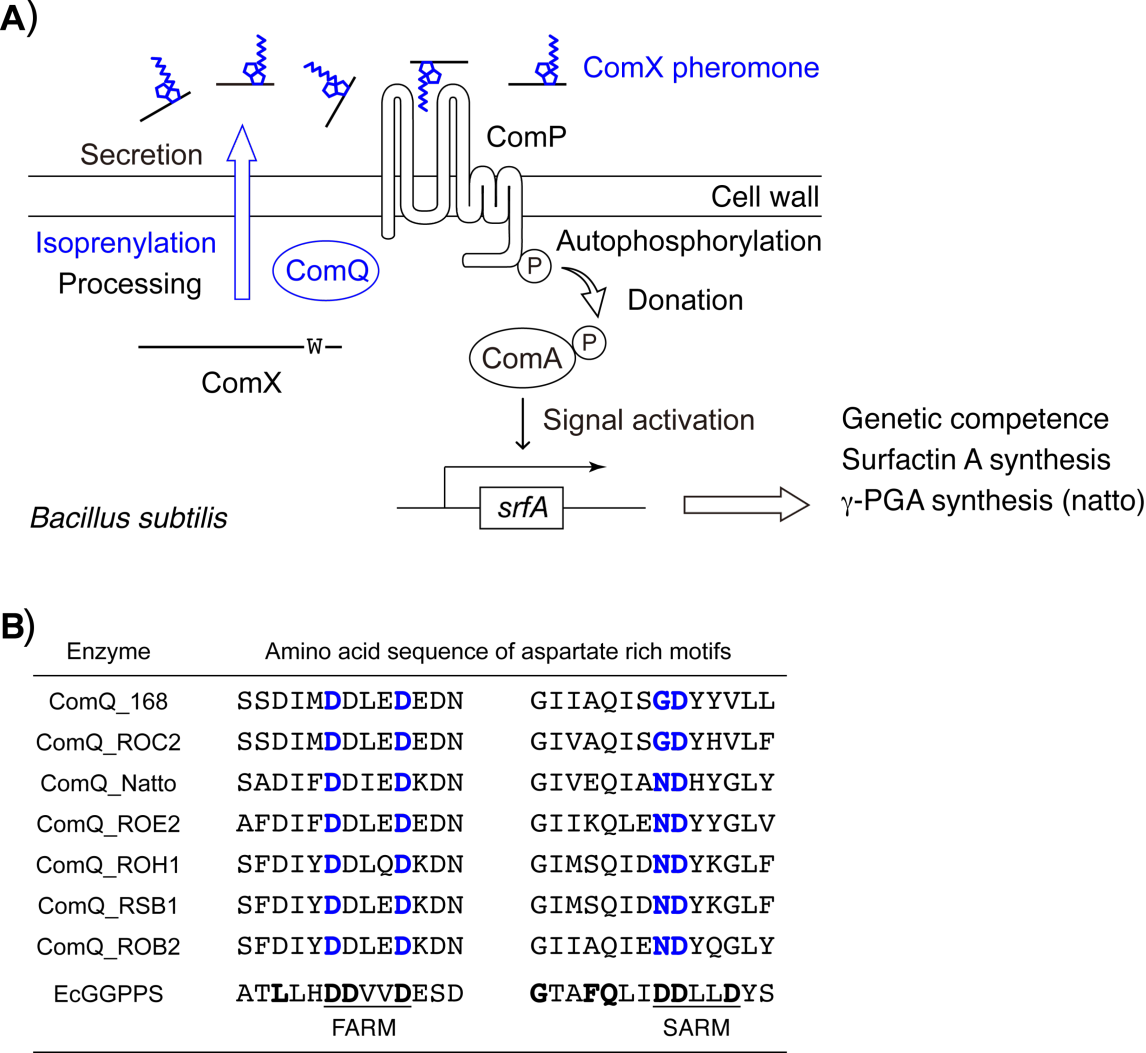
(A) Schematic representation of the signal transduction cascade of quorum sensing stimulated by the ComX pheromone in *B. subtilis*. (B) Amino acid sequences of the aspartate-rich motif and the pseudo aspartate-rich motif in ComQ from seven *Bacillus* strains. Essential amino acid residues for function are shown in bold and colored blue. EcGGPPS is a geranylgeranyl diphosphate synthase derived from *Escherichia coli* ISC56. It’s essential amino acid residues for function are shown in boldface, and the aspartate-rich motifs are underlined.

Molecular genetic analyses of the natural competence of *B. subtilis* revealed that the *comQXPA* gene cluster was responsible for *B. subtilis* to induce the genetic competence involved in the secretion of the ComX pheromone (Figure 4A) [13,21,25]. ComQ, the first protein encoded in the cluster, functions as an isoprenyltransferase for the ComX peptide, which is encoded next in the cluster [35]. The downstream ComP is homologous to transmembrane histidine kinase, and ComA is homologous to a response regulator [36]. Therefore, the two proteins constitute the large family of two-component regulatory systems widely found in bacteria. ComP becomes autophosphorylated in response to the secreted ComX signaling molecule as a receptor, and donates a phosphate group to ComA. The phosphorylated ComA subsequently transmits the signal for activating the surfactin synthase *srfA* operon and mediates the genetic competence in *B. subtilis*. ComQ lacks homology to cysteine isoprenyltransferases, tryptophan dimethylallyltransferases for cyanobactins [2,37–38] or prenyltransferases for indole alkaloids [39–42]. However, ComQ shares some homology with farnesyl diphosphate (FPP) synthases and geranylgeranyl diphosphate (GGPP) synthases, which catalyze the condensation of isopentenyl diphosphate (IPP) with geranyl diphosphate (GPP) or FPP to form C5-extended isoprenyl diphosphates FPP or GGPP (Figure 4B) [43–44]. In the both typical diphosphate synthases, two aspartate-rich motifs containing “DDxxD” residues, in which x refers to any amino acid, are highly conserved. The two “DDxxD” motifs, named the first and second aspartate-rich motifs (FARM and SARM), function as the binding sites for the two substrates, GPP or FPP and IPP, through Mg^2+^ and play a crucial role in the FPP and GGPP syntheses. FARM is also conserved in ComQ, and a previous study demonstrated that the mutation of the first or fifth aspartate of FARM in ComQ to alanine resulted in the elimination of the downstream pheromonal signaling [21]. This result suggested that FARM of ComQ is necessary for the production of the ComX pheromone and possibly functions as a binding site for the extension substrate, GPP or FPP. In contrast to FARM, the amino acid residues corresponding to SARM in ComQ are quite different from those in the typical FPP and GGPP synthases (Figure 4B). Since only the second aspartate is preserved in the corresponding region of ComQ, the region is thus no longer aspartate-rich, and so hereafter it is referred to as a pseudo-SARM. A site-directed mutagenesis analysis of the ComQ_RO-E-2_ from strain RO-E-2 with an in vitro geranylation reaction revealed that the lone-conserved second aspartate residue in the pseudo-SARM of ComQ is also critical for the isoprenylation activity, similar to the second aspartate residue in SARM in the FPP and GGPP synthases [45–46]. In addition, the first amino acid residue of the pseudo-SARM in ComQ, asparagine (or glycine), is crucial for the ComQ function. Particularly, the mutation from asparagine to aspartate drastically decreased the geranylation activity. In contrast, the last three amino acid residues of the pseudo-SARM in ComQ are replaceable, without the loss of ComQ function. Thus, for tryptophan isoprenylation the ComQ must have the sequence NDxxx (or GDxxx) in the pseudo-SARM. Although most FPP and GGPP synthases possess the DDxx(D) amino acid sequence in the SARM, the sequence is unsuitable for the isoprenylation of tryptophan.

### ComX

**Figure 5:**
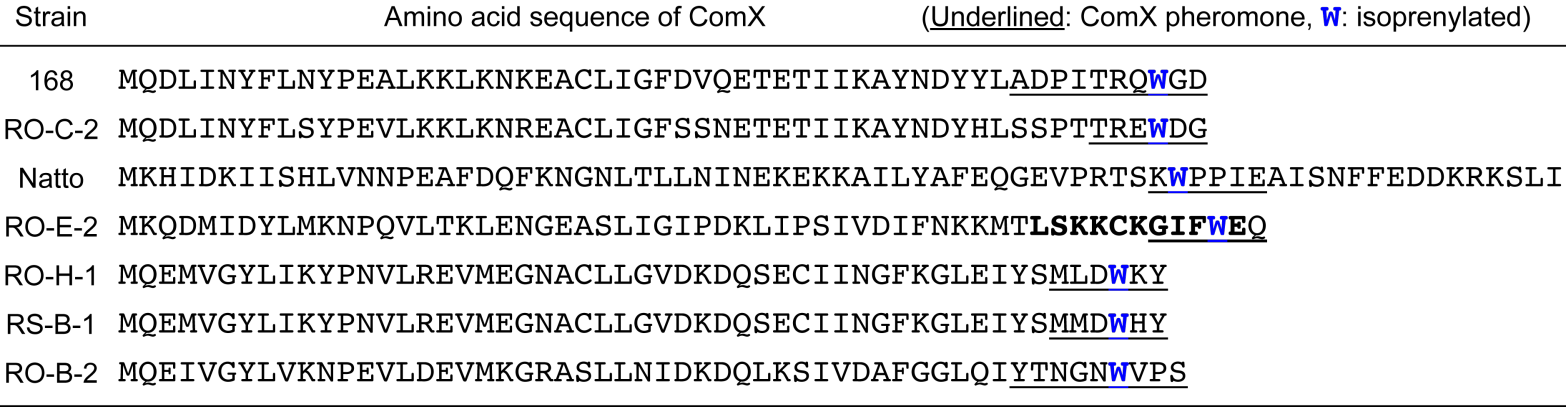
Amino acid sequences of ComX from seven *Bacillus* strains. The sequences of the mature pheromones are underlined, and the isoprenylated tryptophan residues are shown in bold and colored blue.

The ComX precursor peptide possesses 53 to 58 amino acid residues in six *Bacillus* strains, except for subsp. *natto* [25–26]. The tryptophan residue isoprenylated by ComQ is located at either the 3rd or 4th position from the C-terminal end, and the cleavage of the N-terminal residues leads to the production of the mature ComX pheromone with six to ten amino acid residues (Figure 5). In most ribosomally synthesized and posttranslationally modified peptides (RIPPs), a conserved recognition motif in the N-terminal leader region of the precursor peptide enables the enzymatic modification of the C-terminal core peptide, and then the leader amino acids are frequently cleaved [2]. However, there is no obvious sequence within the N-terminal region of the ComX peptide for ComQ recognition, because the truncated C-terminal dodecapeptide of ComX_RO-E-2_ ([47-58]ComX_RO-E-2_, LSKKCKGIFWEQ) showed significant activity for geranyl modification by ComQ_RO-E-2_, although the activity was approximately 10-fold weaker than that of full length ComX_RO-E-2_ [47]. Among the twelve amino acid residues, the N-terminal leucine residue and the modified tryptophan residue were the only conserved amino acids in the ComX variants. Therefore, a common consensus sequence for tryptophan isoprenylation does not seem to exist. In addition, the tryptophan residue modified with a geranyl group must be located at the 2nd, 3rd, or 4th position from the C-terminal end of ComX_RO-E-2_ for geranylation by ComQ_RO-E-2_, based on the in vitro reactions of C-terminal sequence analogs with either a deletion of the two residues or an alanine extension at the C-terminal end. Therefore, the undecapeptide [47-57]ComX_RO-E-2_ from the 47th to the 57th residues of the ComX_RO-E-2_, LSKKCKGIFWE, is the minimum substrate unit for geranylation by ComQ_RO-E-2_. These results are consistent with the fact that the ComX pheromone variants among six *Bacillus* strains possess a modified tryptophan residue at the 3rd or 4th position from the C-terminal end, except for the ComX_natto_ pheromone from subsp. *natto*. Unlike the six ComX pheromone variants, the ComX_natto_ pheromone possesses a modified tryptophan residue with a farnesyl group at the 5th position from the C-terminal end, which corresponds to the 54th residue in the 73 amino acid residues of ComX_natto_; namely, at the 20th position from the C-terminal end. In addition, the C-terminal amino acid residues of ComX_natto_ as well as the N-terminal amino acid residues are processed to form the ComX_natto_ pheromone, corresponding to the 53rd to 58th residues of the ComX_natto_ precursor peptide [24,48]. Although it is presently not clear which step occurs first, the farnesylation of the tryptophan residue or the truncation of the C-terminal amino acid residues, the posttranslational farnesylation was not necessarily limited to a tryptophan near the C-terminus, but also has occurred at an internal tryptophan residue of the precursor peptide.

### Kawaguchipeptin A

**Figure 6:**
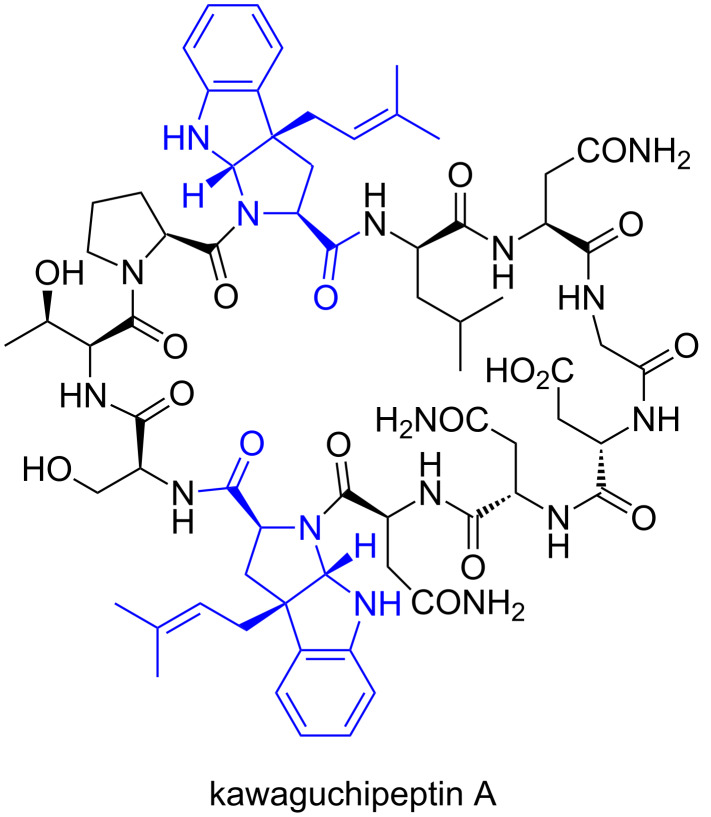
Chemical structure of kawaguchipeptin A. Dimethylallylated tryptophan residues are colored blue.

Apart from the ComX pheromones, post-translational dimethylallylations of the tyrosine, threonine, serine, and tryptophan residues of cyclic peptides from cyanobacteria were reported [49–51]. The RiPPs derived from cyanobacteria, including dimethylallylated cyclic peptides, are called cyanobactins [2,37–38]. Although several cyanobactins exhibit significant biological activities, such as antibacterial and enzyme inhibitory properties, the actual biological role of prenylation in cyanobactins is still unknown at this time. Kawaguchipeptins A and B are members of the cyanobactin family and are macrocyclic undecapeptides with the cyclic amino acid sequence of [WLNGDNNWSTP]. They are produced by *Microcystis aeruginosa* NIES-88 (Figure 6) [52–53]. Kawaguchipeptin A contains one D-leucine and two prenylated tryptophan residues, while kawaguchipeptin B consists only of L-amino acid residues. Interestingly, kawaguchipeptin A possesses two dimethylallylated tryptophan residues, which are modified with a dimethylallyl group at the gamma position, resulting in the formation of a tricyclic structure with the same scaffold as that of the ComX pheromones, but with the opposite stereochemistry [54]. The *KgpA* to *G* gene cluster was identified as encoding the kawaguchipeptins synthase in *M. aeruginosa* NIES-88 [55]. KgpF is a member of the ABBA prenyltransferase family, which shares a common structural motif known as the ABBA fold and exhibits some similarity to other dimethylallyltransferases for cyanobactins and prenyltransferases for indole alkaloids, but lacks similarity to cysteine isoprenyltransferases and ComQs [2,37–44]. Considering the in vitro prenylation analysis of KgpF together with other biosynthetic studies on prenylated cyanobactins, KgpF functions at the end of the biosynthesis, and recognizes two tryptophan residues in the precursor cyclic peptide to form kawaguchipeptin A. In contrast to typical post-translational modifications, a specific amino acid motif adjacent to the core peptide sequence for directing KgpF is unlikely to be required. In addition, the prenylation reaction by KgpF does not seem to need a specific amino acid motif within the core cyclic peptide, because there is no similarity between the sequences surrounding the two tryptophan residues (PWL and NWS) in kawaguchipeptin A. Consistently, KgpF exhibits relaxed substrate specificity toward diverse tryptophan residues in peptides, as KgpF can even accept a single derivatized amino acid, Fmoc-tryptophan, as a substrate and mediate its regioselective and stereoselective dimethylallylation at the C-3 position of its indole ring.

## Conclusion

The posttranslational isoprenylation of tryptophan involving pyrrolidine ring formation was first discovered in a *B. subtilis* peptide pheromone, as a crucial modification for the pheromonal function. In addition, the discovery of the ComX_natto_ pheromone revealed that a tryptophan residue modified with an isoprenyl group is not always restricted to a location near the C-terminal end. The broad substrate tolerance of the modifying enzyme ComQ may attract attention as an enzyme engineering target for the synthesis of prenylated tryptophan derivatives. However, since the consensus sequences for tryptophan isoprenylation in the ComX precursor peptide and the ComX pheromone homologues have yet to be identified, it is presently considered that the post-translational geranylation or farnesylation of tryptophan is a special modification in several *Bacillus* species. In contrast, numerous peptides and proteins post-translationally modified with farnesyl or geranylgeranyl groups on the cysteine residues were identified in a variety of organisms. However, the isoprenylation was not considered to be universal at first. The isoprenylation of cysteine was also first found in peptide pheromones from a specific microorganism, as an essential modification for the pheromonal activity. Thus, it is conceivable that the posttranslational isoprenylation of tryptophan is actually widespread. Therefore, more research should be focused on the details and the diversity of the post-translational isoprenylation of tryptophan.
